# Coherent acoustic phonon dynamics in chiral copolymers

**DOI:** 10.1063/1.5124438

**Published:** 2019-12-23

**Authors:** Mirko Scholz, Marius Morgenroth, Min Ju Cho, Dong Hoon Choi, Thomas Lenzer, Kawon Oum

**Affiliations:** 1 Physical Chemistry, University of Siegen, Adolf-Reichwein-Str. 2, 57076 Siegen, Germany; 2 Department of Chemistry, Research Institute for Natural Sciences, Korea University, 5 Anam-dong, Sungbuk-gu, Seoul 136-701, South Korea

## Abstract

Coherent phonon oscillations in the UV-Vis transient absorption and circular dichroism response of two chiral polyfluorene-based copolymer thin films are investigated. A slow oscillation in the hundred picosecond regime indicates the propagation of a longitudinal acoustic phonon with a frequency in the gigahertz range through cholesteric films of PFPh and PFBT, which allow for the optical determination of the longitudinal sound velocity in these polymers, with values of (2550 ± 140) and (2490 ± 150) m s^−1^, respectively. The oscillation is induced by a strain wave, resulting in a pressure-induced periodic shift of the electronic absorption bands, as extracted from a Fourier analysis of the transient spectra. The acoustic phonon oscillation is also clearly detected in the transient circular dichroism (TrCD) response of PFPh, indicating a transient pressure-induced shift of the CD spectrum and possibly also phonon-induced chirality changes via pitch length modulation of the cholesteric helical polymer stack.

## I. INTRODUCTION

Picosecond ultrasonics^1^ has become a powerful tool for the laser-based, contactless investigation of thin films and nanostructures covering the frequency range from a few to several hundred gigahertz.^2–10^ In a system of interest, e.g., a metal, semiconductor, or polymer thin film, acoustic phonons are generated either directly by an ultrashort laser pulse^11–13^ or by exciting an attached transducer layer,^14–17^ which launches an acoustic pulse in the adjacent film structure. Such all-optical nanoultrasonics^8^ has found widespread applications, and recent examples include buried nanointerfaces, multiple quantum wells, or organic-inorganic heterostructures.^10,16,18,19^ Previous optical investigations largely employed detection at a single or a few probe wavelengths in order to extract information regarding longitudinal and transversal (shear) acoustic waves in various thin film materials, and many of these measurements have been performed in reflective geometry.^4,6,11,16,17,20^

**FIG. 1.**
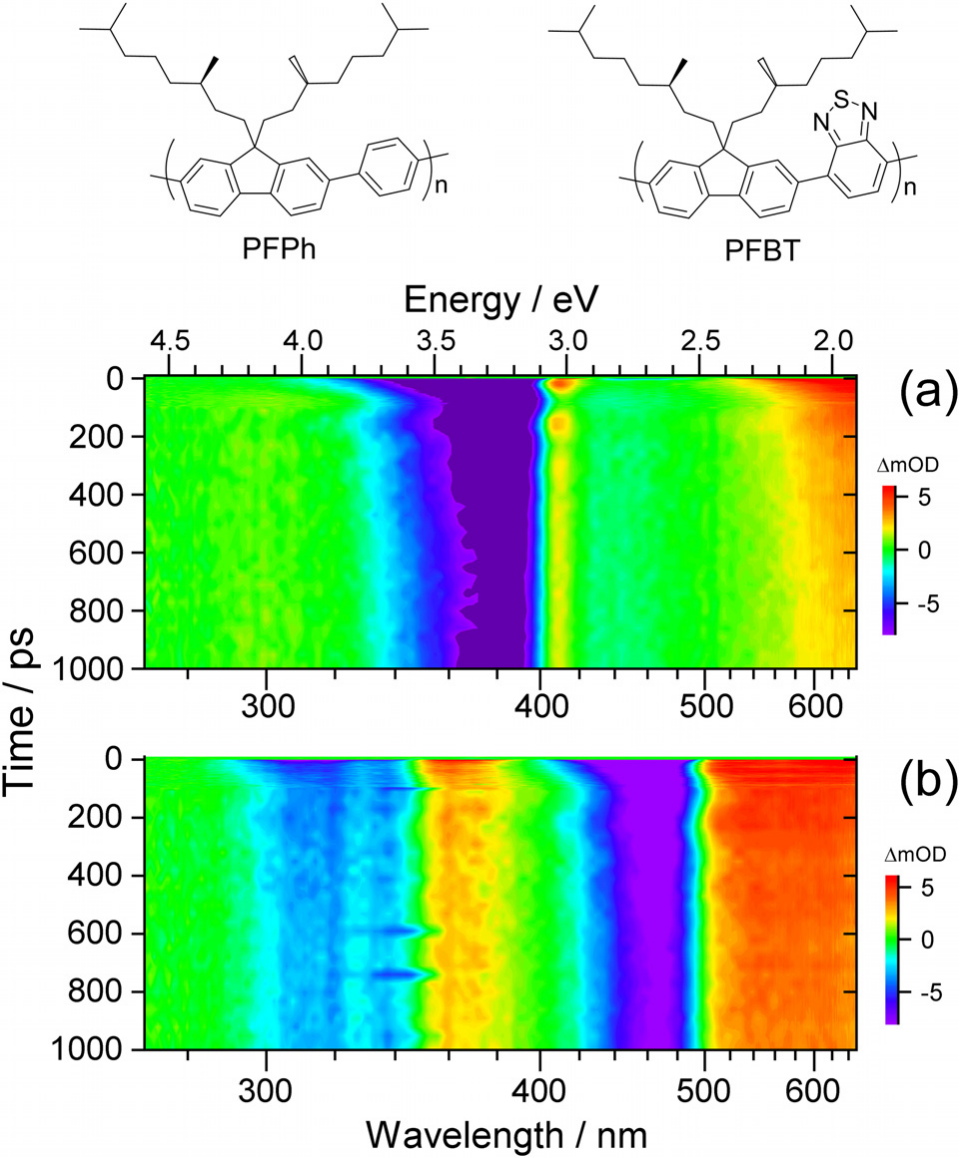
Contour maps of UV-Vis broadband transient absorption spectra for (a) PFPh and (b) PFBT thin films. The chemical structures of the two polyfluorene-based copolymers are shown on top.

In this contribution, we investigate the viscoelastic response of thin nanolayers of two previously unstudied chiral polyfluorene copolymers denoted as PFPh and PFBT (see top of Fig. 1 for their chemical structures) using ultrafast coherent acoustic phonon spectroscopy in transmission mode. Based on the evaluation of the complete spectrotemporal datasets from broadband transient absorption, we aim at determining accurate values for the previously unknown longitudinal speed of sound inside these polymer compounds and obtaining a clear picture of the physical processes responsible for the oscillatory optical response.

Moreover, further technical developments in the field of picosecond ultrasonics are highly desirable to obtain additional information regarding the structural dynamics in such films. As a new step in this direction, we apply time-resolved circular dichroism spectroscopy to detect changes in chirality induced by the coherent acoustic phonons. We indeed find indications for such transient structural modifications of the chiral polymer arrangement.

## II. METHODS

### A. Sample preparation and characterization

The synthesis and characterization of the chiral PFPh and PFBT were described recently.^21^ Cholesteric copolymer thin films were produced by spin-coating a filtered 7.5 mg ml^−1^ solution of each polymer in a chlorobenzene-chloroform mixture (1:9 by volume) on thoroughly cleaned and ozonized Duran glass slides (thickness 1 mm) for one minute at 1000 or 500 rpm, respectively. Films were subsequently annealed between 90 and 120 °C for 15 min. The thickness of the films was determined by AFM using a PSIA XE-100 atomic force microscope. The measurements were carried out in “no contact mode” using a silicon tip. Films were scratched by a pair of fine-tipped stainless steel tweezers. Over a length of 64 and 80 *μ*m, 64 data points were recorded at a scan rate of 0.05 Hz for PFPh and PFBT, respectively.

### B. Broadband transient absorption and time-resolved CD spectroscopy

Broadband transient absorption spectroscopy of the PFPh and PFBT thin films was performed using the pump-supercontinuum probe (PSCP) method^22^ and carried out on a setup covering the UV-Vis (260–700 nm) range employing single-shot referencing.^23^ The time resolution was ca. 60 fs. Ultrafast transient circular dichroism (TrCD) spectra of PFPh were obtained on a setup, which was described in detail previously.^24^ Briefly, a multifilament supercontinuum was generated using 400 nm seed pulses of either left-handed or right-handed circular polarization. The polarization state was switched by a BBO Pockels cell. The TrCD signal was obtained from four consecutive measurements as ΔCD = ΔΔOD = ΔOD_L_ − ΔOD_R_, where ΔOD_L_ and ΔOD_R_ are the difference optical densities with and without pump beam for left-handed and right-handed circular polarization. The time resolution of this TrCD setup was ca. 170 fs. The excitation wavelength for both types of experiments was 370 nm. The pump fluence was 230 *μ*J cm^−2^ for PFPh and 700 *μ*J cm^−2^ for PFBT. Thin-film steady-state absorption spectra were measured using a Varian Cary 5000 dual-beam spectrophotometer.

The transient absorption spectra of PFPh and PFBT were subjected to a Fourier transformation analysis as previously described.^25,26^ Briefly, the pure oscillatory contribution to the experimental spectrum was obtained by subtracting a global monoexponential fit from the experimental data. All time traces in the respective dataset were then zero-padded to four times their original length and multiplied with an exponential-decay apodization function. Fourier transformation of the modified experimental time trace provided the frequency magnitude. Frequency spectra were obtained by averaging over selected wavelength intervals.

## III. RESULTS AND DISCUSSION

### A. Overview of UV-Vis transient absorption spectra for PFPh and PFBT

Figure 1 shows contour plots of the UV-Vis transient absorption spectra for PFPh and PFBT thin films in panels (a) and (b), respectively. Violet and blue regions indicate ground state bleach (GSB) or stimulated emission (SE), whereas yellow to red colors are due to excited state absorption (ESA). Green areas correspond to spectral regions with only weak transient changes. Briefly, the 370 nm pump pulse excites both copolymers to the S_1_ state. As a result, PFPh shows a single GSB/SE feature centered at 380 nm (S_0_ → S_1_). PFBT exhibits a stronger GSB (S_0_ → S_1_) plus SE (S_1_ → S_0_) feature at 460 nm and a weaker band (GSB, S_0_ → S_n_) at 320 nm, respectively. S_1_ ESA bands are observed at 410 and 620 nm for PFPh and at 360 and 590 nm in the case of PFBT.

The subsequent dynamics in both polymers involves intrachain and interchain exciton migration as well as exciton recombination, but this is not in the center of the current investigation. Instead, we focus on slow oscillatory features present in both datasets. These slow damped oscillations are clearly visible for PFPh in terms of periodically modulated GSB and ESA in the 390–420 nm range and modulated GSB in the 320–360 nm region. In the case of PFBT, four regions showing oscillations are apparent: the GSB/SE/ESA region at 480–520 nm as well as the GSB region at 400–440 nm, both connected with the S_0_ → S_1_ band, and the GSB/ESA range 290–360 nm in the region of the S_0_ → S_n_ band. A complete analysis will be provided in the following.

### B. Coherent acoustic phonon oscillations in transient absorption spectra

**FIG. 2.**
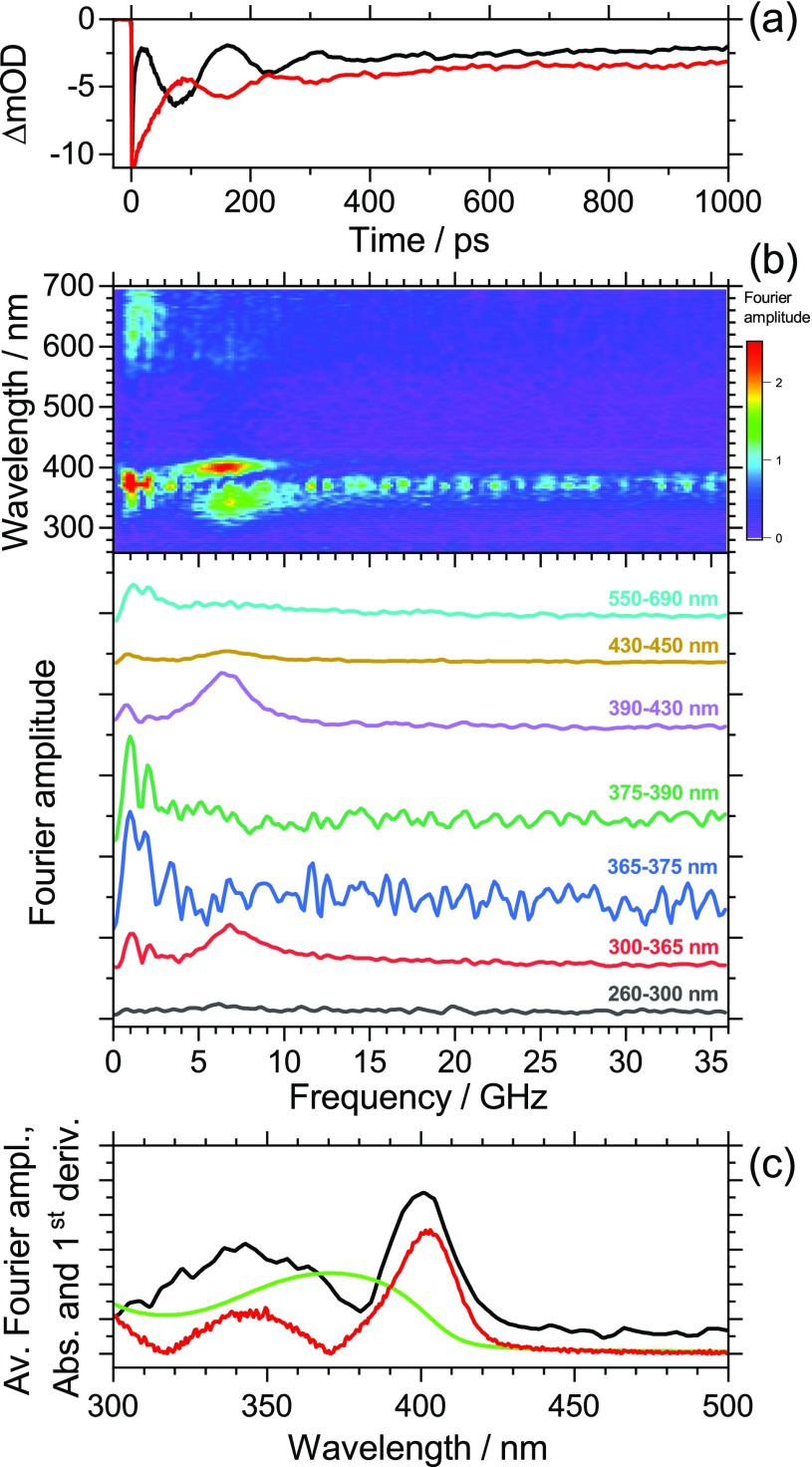
(a) Kinetics of a PFPh thin film averaged over the wavelength ranges 395–405 nm (black line) and 338–348 nm (red line) showing damped oscillations arising from a coherent longitudinal acoustic phonon. Note the phase shift of π for the two kinetics. (b) Contour map of Fourier amplitudes extracted from the complete spectral dataset (top) and representative averaged spectral slices from the contour map for the wavelength ranges indicated (bottom). The slices are vertically shifted to avoid overlap of the traces. The noise of the slice for 365–375 nm is slightly increased due to pump-beam stray light. The slices show the pronounced contribution of a coherent acoustic phonon at 7.0 GHz particularly in the wavelength ranges 300–365 and 390–430 nm. (c) Wavelength-dependent Fourier amplitude averaged over the frequency interval (7.0 ± 0.9) GHz (black line), steady-state absorption spectrum of PFPh (green line) and absolute value of the first derivative of the steady-state absorption spectrum (red line).

Figure 2(a) shows selected kinetics for PFPh, averaged over the wavelength ranges 338–348 (red line, low-wavelength shoulder of the S_0_ → S_1_ absorption band) and 395–405 nm (black line, high-wavelength shoulder of the S_0_ → S_1_ absorption band). The period of the oscillation is *τ*_a_ = 143 ps. The two kinetic traces exhibit a phase shift of π. For a detailed interpretation of the features, the complete set of transient absorption spectra was analyzed by Fourier transformation in order to extract the wavelength-dependent oscillatory contributions. The resulting contour map of the Fourier amplitudes is presented in Fig. 2(b), including spectral slices for the wavelength intervals indicated. We observe two strong peaks centered at 343 and 400 nm. The corresponding oscillation has a frequency of 7.0 GHz. The low frequency clearly identifies this mode as a coherent acoustic phonon. It is induced by the ultrashort 370 nm pump pulse, which launches a longitudinal sound wave inside the PFPh polymer thin film, i.e., a strain wave packet propagating ballistically through the polymer.^3,27^ Contributions of intra- and interchain vibrations of the polymers can be excluded because these frequencies are typically larger than 1 THz, as determined from neutron inelastic scattering.^28^

The acoustic pulse bounces back and forth at the film boundaries, which induces a periodic modulation of the transient optical response.^1,4,5,9,29^ Our measurement provides an elegant and simple means to determine the longitudinal speed of sound *c*_L_ inside the copolymer film, which is given by^4^

$$c_{L}=4d/\tau_{a}.$$ (1)

Here, *d* is the thickness of the film, which was determined as (91.2 ± 3.6) nm from AFM experiments. Using *τ*_a_ = (143 ± 2) ps for the period of the oscillation, we arrive at *c*_L_ = (2550 ± 140) m s^−1^ for the longitudinal speed of sound in PFPh at 296 K.

The value for this polyfluorene-based copolymer may be compared with sound velocities determined previously for other polymers, such as polyethylene (2430 m s^−1^), polypropylene (2650 m s^−1^), polymethylmethacrylate (2690 m s^−1^), and polystyrene (2400 m s^−1^).^30^ We note that by knowing this value for *c*_L_, metrology of PFPh thin films with nanometer resolution in a noninvasive manner is now possible by simply measuring the oscillation period in the transient PSCP kinetics and applying Eq. (1).

Furthermore, in Fig. 2(a), we observe a characteristic damping of the oscillations. A global fit to the data provides a damping time constant of *τ*_d_ = 154 ps, which is very similar to the oscillation period of *τ*_a_ = 143 ps. For normal incidence and ideal mechanical contact, the intensity reflection coefficient *R*_12_ of an acoustic wave at an interface can be determined from the acoustic impedances *Z*_1_ and *Z*_2_ of the two media as

$$R_{12}={\left(\frac{Z_{1}-Z_{2}}{Z_{1}+Z_{2}}\right)}^{2},\;\text{with}\;Z_{i}=\rho_{i}\;c_{L,i}.$$ (2)

Here, *ρ_i_* and *c*_L,__*i*_ are the density and the speed of sound in the two media, respectively. Using typical values for a polymer/nitrogen interface [*ρ*_nitrogen_ = 1.138 kg m^−3^ (ideal gas), *c*_L,nitrogen_ = 350.8 m s^−1^,^31^ *ρ*_polymer_ = 880 kg m^−3^ (polyfluorene PF8),^32^ and *c*_L,polymer_ = 2550 m s^−1^ (PFPh)], one obtains the reflectivity *R*_12_(polymer/nitrogen) = 100%, which is the “zero-stress boundary condition” for a free surface.^4^ Therefore, significant attenuation of the sound wave only occurs at the glass/polymer interface, having an “approximately zero displacement boundary condition.”^4^ Because the measured damping time constant is close to the measured oscillation period, i.e., *τ*_d_ ≈ *τ*_a_, the “experimentally determined reflectivity” is about 1/*e* (= 37%). This value may be compared with an estimate based on Eq. (2) employing values for a typical Duran borosilicate glass (*ρ*_glass_ = 2220 kg m^−3^, *c*_L,glass_ = 5252 m s^−1^),^33,34^ providing *R*_12_(polymer/glass) = 45.8%, which is slightly larger, but in reasonable agreement. In addition, a minor contribution of acoustic attenuation by the polymer material could account for the difference between the measured and calculated value.^5,29,35^

Next, we would like to comment on the mechanism responsible for the modulation of the optical response. Figure 2(c) shows the wavelength-dependent Fourier amplitude averaged over the frequency interval (7.0 ± 0.9) GHz (black line). This Fourier spectrum closely resembles the absolute value of the first derivative (red line) of the steady-state absorption spectrum (green line). Therefore, the largest amplitude of the oscillation is found on the wings of the S_0_ → S_1_ absorption band, whereas this oscillation vanishes at the band maximum. Recognizing that the kinetics on the low-wavelength and high-wavelength wings exhibit a phase shift of π [Fig. 2(a)], the observed spectral behavior is consistent with a transient periodic shift of the S_0_ → S_1_ absorption band. From the amplitude of the oscillation on the red wing of the band (ca. 6 × 10^−3^ at 3.082 eV) and the slope of the absorption spectrum of 3.5 eV^−1^ we estimate a maximum red shift of 1.7 meV. The following generation mechanism of the periodic band shift is suggested: photoexcitation of PFPh occurs near the polymer/glass interface. In the present case, 50% of the pump photons are already absorbed within the first 40 nm of the film. Because the lateral dimension of the pump pulse (300 *μ*m) is much larger than the film thickness (91.2 nm), this propagation can be treated as a one-dimensional problem. Photoexcitation to S_1_ has the following effects: the electronic excitation leads to changes in the bond lengths and the electron distribution of the polymer. This “deformation” leads to the emission of an acoustic phonon (“deformation potential mechanism”).^3^ In addition, fast exciton relaxation and recombination quickly releases a substantial amount of energy into the polymer film,^11^ which induces fast thermal heating on the picosecond time scale and expansion of the polymer, again launching an acoustic phonon.^3,36^ These two mechanisms result in a rapid “pressure increase” due to the formation of a pump-beam-induced strain pulse, leading to an initial ultrafast red shift of the absorption band. Pressure-induced red shifts of polymer steady-state absorption spectra are well known.^35,37^ Also, a transient analog of this “pressure effect” has been observed previously in dynamic Stokes shift measurements for small organic molecules.^38^ In that case, this process was linked to an ultrafast shrinkage of the “solute cavity.” For a thin film, this is equivalent to an increase in the repulsive forces between the excited chromophore and the surrounding polymer chains upon photoexcitation, inducing strain in the polymer. The length of the acoustic pulse can be approximated as the ratio of the depth *d* of the “stressed region” and the longitudinal sound velocity *c*_L_.^3^ Assuming typical values of 40 nm and 2500 m s^−1^ one obtains an acoustic pulse length of 16 ps. This acoustic sound wave then propagates through the polymer and bounces back and forth between the two interfaces, and damping occurs at the polymer/glass interface, as described above. We also note that there could be additional contributions to the periodic shift of the absorption band due to a dynamic Stark shift.^39^ Photoexcitation produces electrons and holes, which generate an electric field in the polymers. The strain induced by the propagating acoustic phonon could then modulate the field strength and thus the band positions in the absorption spectra.

**FIG. 3.**
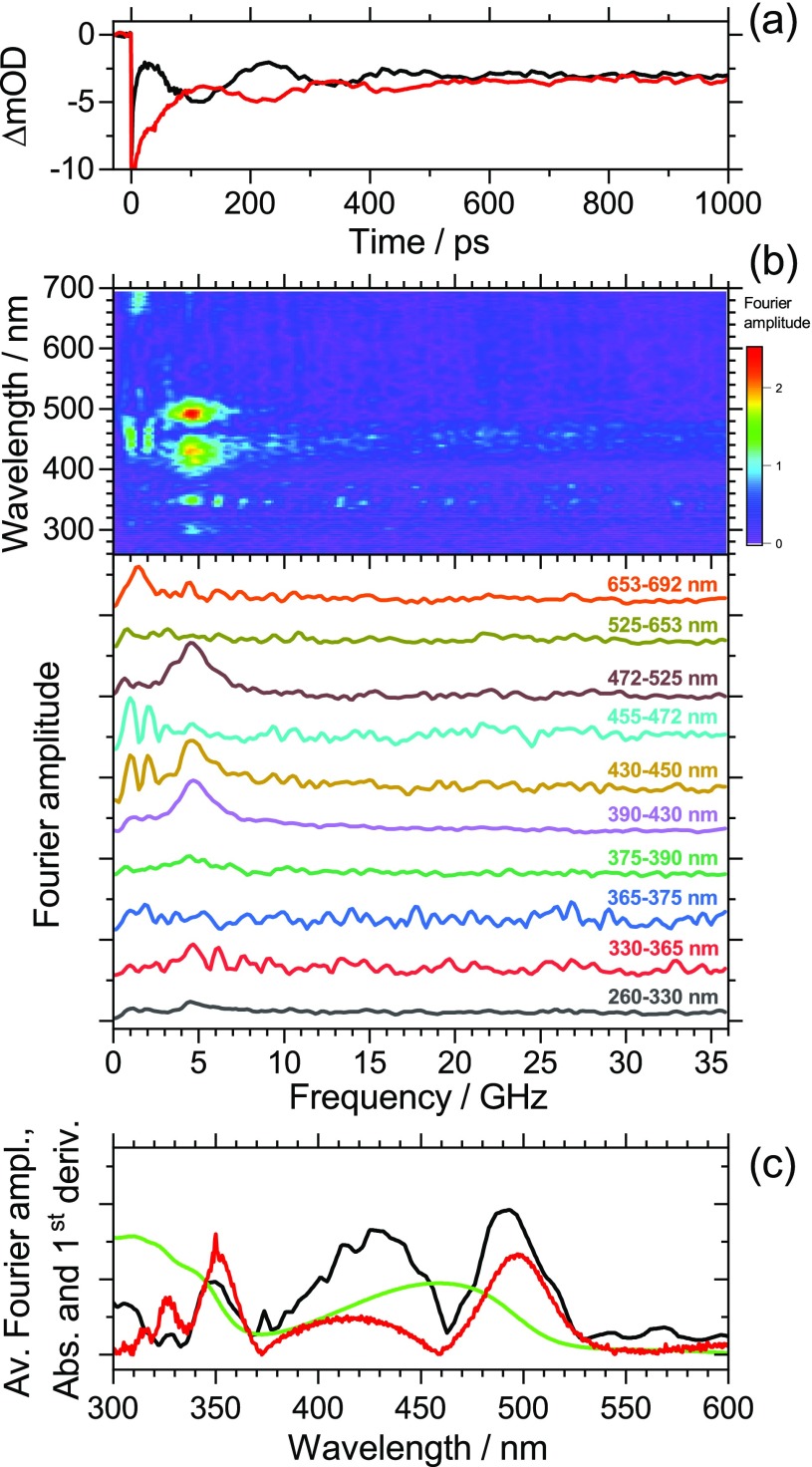
(a) Kinetics of a PFBT thin film averaged over the wavelength ranges 485–495 nm (black line) and 424–434 nm (red line) showing damped oscillations arising from a coherent longitudinal acoustic phonon. A phase shift of π is observed as for PFPh. (b) Contour map of Fourier amplitudes extracted from the complete spectral dataset (top) and representative averaged spectral slices from the contour map for the wavelength ranges indicated (bottom). The slices are vertically shifted to avoid overlap of the traces. The noise of the slice for 365–375 nm is slightly increased due to pump-beam stray light. The slices show the pronounced contribution of a coherent acoustic phonon at 4.6 GHz particularly in the wavelength ranges 390–450 and 472–525 nm. (c) Wavelength-dependent Fourier amplitude averaged over the frequency interval (4.6 ± 0.9) GHz (black line), steady-state absorption spectrum of PFBT (green line) and absolute value of the first derivative of the steady-state absorption spectrum (red line).

A corresponding investigation was carried out for PFBT thin films. The results are presented in Fig. 3. The peak of the S_0_ → S_1_ absorption band of PFBT is located at 458 nm (2.705 eV). As in the case of PFPh, the averaged kinetics in panel (a) for the red wing (485–495 nm, black line) and the blue wing of the band (424–434 nm, red line) show a pronounced oscillation with a phase shift of π. The period of the oscillation is *τ*_a_ = 215 ps. The complete set of transient absorption spectra in the range 260–700 nm was again subjected to a Fourier transform analysis. The resulting contour map of Fourier amplitudes including selected spectral slices is presented in panel (b). We observe strong peaks at 429 and 492 nm and weak peaks at 296 and 350 nm. The corresponding oscillation has a frequency of 4.6 GHz. As in the case of PFPh, we assign it to a low-frequency coherent acoustic phonon induced by the ultrashort 370 nm pump pulse. Figure 3(c) shows the wavelength-dependent Fourier amplitude averaged over the frequency interval (4.6 ± 0.9) GHz (black line). As in PFPh, this Fourier spectrum is very similar in shape to the absolute value of the first derivative (red line) of the steady-state absorption spectrum (green line). We also note that the peak pairs 429/492 nm and 296/350 nm both show a phase shift of π, indicating the same type of periodic shift on both absorption bands. The oscillation on the red wing of the S_0_ → S_1_ band (ca. 5 × 10^−3^ at 2.494 eV) and the slope of the absorption spectrum of 2.2 eV^−1^ lead to a maximum red shift of 2.3 meV. As for PFPh, the mechanism therefore appears to a transient “pressure increase” due to the pump-beam-induced strain pulse.

We note that in Fig. 3(c), there are differences between the Fourier amplitude spectrum (black line) and the absolute value of the first derivative of the steady-state absorption spectrum (red line), e.g., for the “red-edge features” at 492 and 350 nm. According to a theoretical study of the closely related polymer F8BT, the absorption band at 458 nm has substantial charge-transfer (CT) character, whereas the band rising toward 300 nm is assigned to a π-π* transition.^40^ Because of the different electronic character, different pressure-induced spectral shifts are expected for the two bands, which easily explains the amplitude differences observed in Fig. 3(c). Obviously, the pressure-induced band shift is smaller for the UV absorption band.

**FIG. 4.**
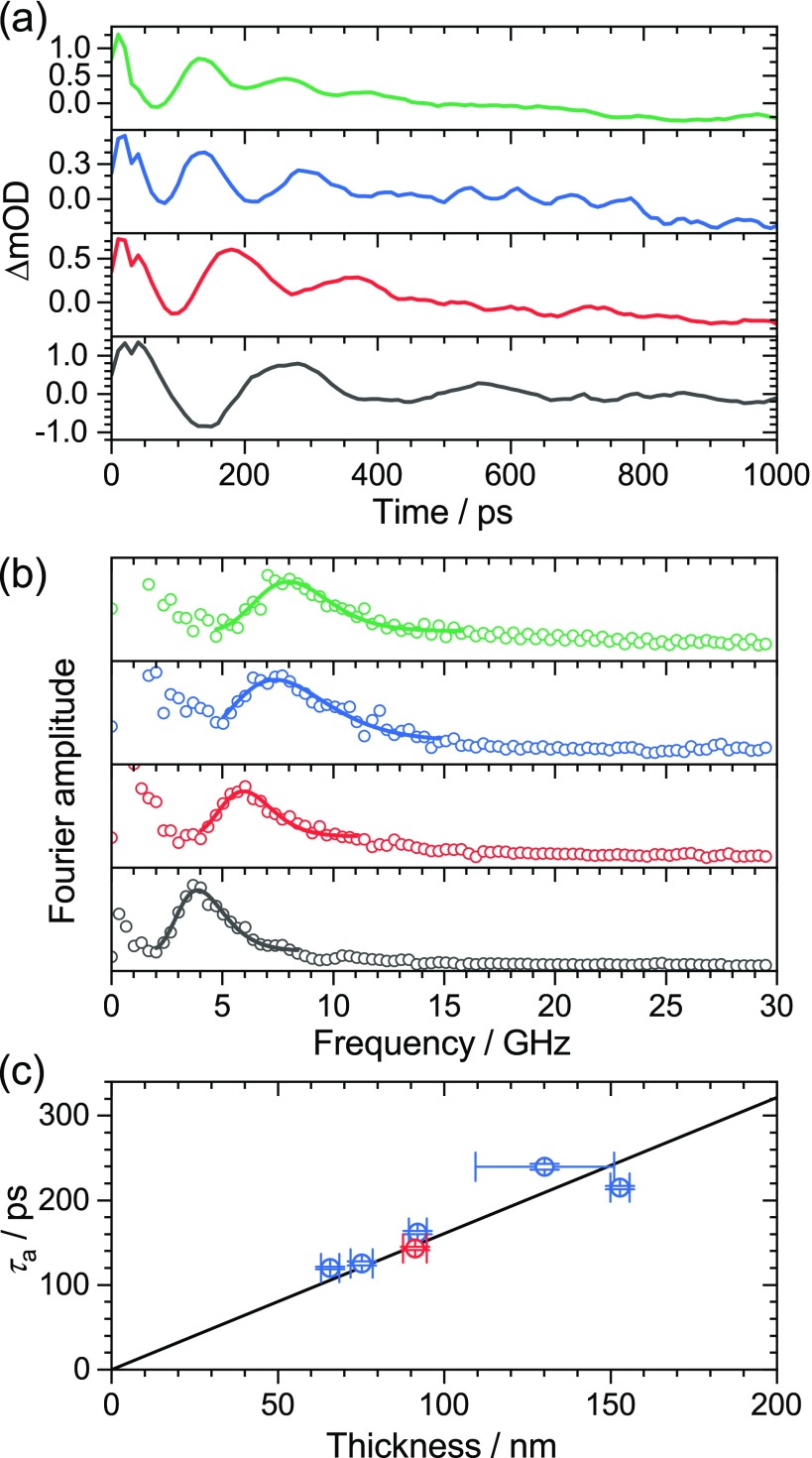
(a) Transient absorption kinetics for PFBT polymer thin films of different thickness averaged over the wavelength range 458–527 nm showing damped oscillations with different period. From bottom to top: spin-coating at 500 rpm (black), 1000 rpm (red), 2000 rpm (blue) and 4000 rpm (green). (b) Fourier amplitude spectra obtained from the transient absorption datasets of the four polymer films. The lines represent lognormal fits to the data for extracting the frequencies of the acoustic phonon. Same color coding as in panel (a). (c) Plot of the oscillation period *τ*_a_ vs the film thickness including error bars. The thickness was determined by AFM experiments. Blue points: results for PFBT, red point: result for PFPh. The black line is a linear fit to the data for PFBT (blue points) with a forced intercept of zero. The resulting slope is (1.608 ± 0.097) ps nm^−1^.

Furthermore, we determined the longitudinal sound velocity *c*_L_ for a series of PFBT polymer thin films having different thickness employing PSCP and AFM experiments. The thickness was varied by a systematic variation of the rotational speed during the spin-coating process, using values in the range of 500–4000 rpm. This resulted in a systematic decrease in the film thickness with increasing rotational speed as confirmed by AFM measurements. Representative results of these experiments are shown in Fig. 4. Panel (a) shows transient absorption kinetics of the four films, which were averaged over the wavelength range of 458–527 nm. We observe a systematic decrease in the oscillation period *τ*_a_ with decreasing film thickness *d*. This proportionality between *τ*_a_ and the thickness *d* of the polymer film is expected, according to Eq. (1). The four transient absorption datasets were then subjected to a Fourier-transform analysis. This provided the Fourier amplitude spectra in Fig. 4(b). The main peak in each spectrum corresponds to the frequency of the longitudinal acoustic phonon. The frequency of the acoustic phonon increases with the decreasing thickness of the polymer films.

In panel (c), we provide a plot of the oscillation period *τ*_a_ vs the film thickness, as determined by AFM. Error bars for *τ*_a_ were derived from the lognormal fits of the Fourier amplitudes in panel (b). The error bars for the thickness were extracted from the AFM measurements. Typically, several lines were scratched into the polymer films. For each of these lines, several measurements were performed and averaged. It turned out that the variation in thickness along one line was smaller than between different lines. In particular, one of the films with the slowest rotation speed of 500 rpm showed significant variation in the thickness, whereas the films spin-coated at higher speeds were uniform [see panel (c)]. According to Eq. (1), we fitted the data by linear regression with a forced intercept of zero. This resulted in a reasonable fit (black line) with a slope of (1.608 ± 0.097) ps nm^−1^. From that slope, we obtain a value of *c*_L_ = (2490 ± 150) m s^−1^ for the longitudinal speed of sound in PFBT at 296 K, in good agreement with the result for PFPh (red point in Fig. 4(c)). Obviously, the replacement of the phenyl by a benzo[c][1,2,5]thiadiazole unit has only a minor impact on the sound velocity in the copolymer. The damping constants *τ*_d_ for the oscillations of PFBT are again similar to the oscillation periods *τ*_a_. This suggests that the experimental reflectivity is approximately 1/*e* (37%) as for PFPh. Using our measured value *c*_L_ = 2490 m s^−1^ and the aforementioned material properties for polymer and glass, we obtain *R*_12_ = 46.8% from Eq. (2), again in satisfactory agreement with the estimate based on the time constants from the PSCP experiment. Table I provides a summary of the characteristic properties for the polymers employed in this study.

**TABLE I.** Properties of PFPh and PFBT copolymer thin films prepared in this study.

Polymer	*υ* (rpm)^a^	*d* (nm)^b^	*τ*_a_ (ps)	*ν* (GHz)	*c*_L_ (m s^-1^)	*R*_12_ (%)^c^
PFPh	500	91.2 ± 3.6	143 ± 2	7.0 ± 0.1	2550 ± 140	45.8
PFBT	500	152.8 ± 2.9	215 ± 2	4.6 ± 0.1	2490 ± 150^d^	46.8
	500	130 ± 21	240 ± 3	4.2 ± 0.1		
	1000	92.0 ± 2.7	162 ± 2	6.2 ± 0.1		
	2000	75.2 ± 3.3	126 ± 3	8.0 ± 0.2		
	4000	65.7 ± 2.7	120 ± 2	8.3 ± 0.1		

^a^ Rotational speed employed for spin-coating of the polymer films.

^b^ Thickness of polymer thin films determined from AFM measurements.

^c^ Intensity reflection coefficient for the interface between the polymer and borosilicate (Duran) glass.

^d^ From the fit to the five data points for PFBT.

We would like to address a few additional points relevant to the coherent phonon dynamics of PFPh and PFBT. First, the coherent phonon oscillations observed here are insensitive to the pump wavelength. This was confirmed by experiments employing excitation at 400 nm, which provided the same oscillation period. In addition, the modulation of the kinetic traces is more pronounced at higher pump laser fluence. These results suggest that it is mainly important that a sufficient number of photons is absorbed in the sample. This way a sufficient local strain in the polymer can be built up to launch the acoustic phonon. In that respect, it is also advantageous if the absorption length in the polymer is short (i.e., that the absorption coefficient is high), so that the laser power is absorbed in a relatively small volume of the polymer close to the glass-polymer interface.

In addition, we would like to comment on low-frequency features observed for both polymers at about 1.5 GHz. In the case of PFPh [Fig. 2(b)], the strongest part of this low-frequency feature is located at 370 nm (peak of the S_0_ → S_1_ absorption band). However, there is also substantial noise from pump-light scatter, as can be seen over the whole frequency range at that wavelength. A weaker feature having about the same frequency is seen in the wavelength range of 600–700 nm. In the case of PFBT [Fig. 3(b)], there are similar weak features at 450 nm (S_0_ → S_1_ peak absorption) and above 680 nm, in that case both not disturbed by scattered pump light. The frequency of 1.5 GHz is, by a factor of 3–5, lower than the longitudinal acoustic phonon frequency. Such a value would be consistent with a transversal (shear) wave.^30,41^ The shear wave could be excited by the 370 nm pump beam, which is entering the polymer surface at an angle of about 10° relative to the surface normal. However, one should be cautious with such an interpretation. This low frequency corresponds to a period of 600–700 ps, i.e., only two full cycles are covered within the 1500 ps time window of our experiments. For instance, a weak up-and-down drift in pump laser power could lead to a similar but spurious low-frequency peak after Fourier transformation.

### C. Coherent acoustic phonon oscillation in transient circular dichroism kinetics

**FIG. 5.**
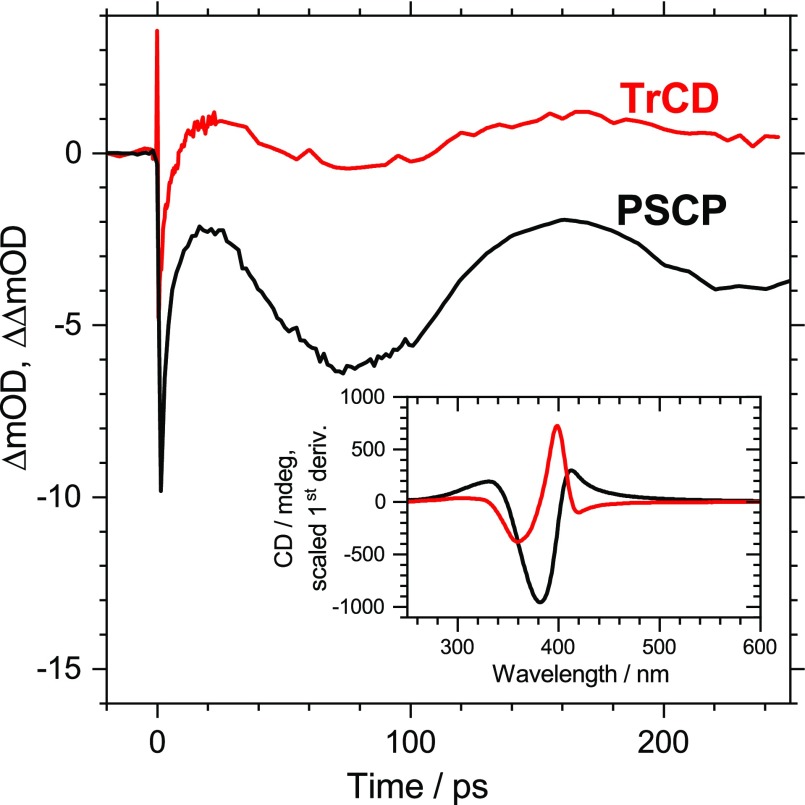
Transient circular dichroism kinetics (red line) and pump-supercontinuum probe transient absorption kinetics (black line) of the PFPh thin film averaged over the wavelength range 395–405 nm. The steady-state CD spectrum of PFPh (black line) and its scaled smoothed first derivative (red line) are shown in the inset. Note that clear oscillatory contributions to the TrCD response are found at wavelengths close to the maximum signal of the first derivative of the CD spectrum.

In addition, we carried out transient circular dichroism measurements on the cholesteric PFPh thin film. TrCD and transient absorption kinetics averaged over the wavelength range of 395–405 nm are compared in Fig. 5. Interestingly, the same oscillation already observed in the transient absorption measurements also persists in the TrCD experiments. The large absolute amplitude of the steady-state CD signal of the PFPh polymer thin film (ca. 1000 mdeg, see the black line in the inset of Fig. 5)^21,42^ arises from the cholesteric arrangement of the polymer chains.^43–45^ The CD spectrum of the polymer will also experience a pressure-induced periodic shift because of the propagation of the acoustic phonon inside the film. This explains why the same type of oscillation is observed in the transient CD spectrum. Clear oscillations are found in the wavelength range close to the maximum of the first derivative of the steady-state CD spectrum (red line in the inset of Fig. 5). In addition, it is known, that steady-state CD signals of such cholesteric systems are very sensitive to the pitch length of the helical stack.^43^ Therefore, the transient CD response in Fig. 5 might also contain a substantial contribution from the periodic compression/extension of the helical stacks in the film, which is induced by the propagating acoustic phonon.

## IV. CONCLUSIONS

Our investigation using coherent phonon spectroscopy has provided a clear picture of the propagation of photoinduced acoustic phonons through two polyfluorene-based copolymer thin films. Coherent oscillations in the transient absorption spectra are assigned to a periodic shift of the ground state absorption bands of the polymers due to a propagating strain pulse. The longitudinal speed of sound for the two polymers is about 2500 m s^−1^ at 296 K, based on a detailed Fourier transformation analysis of the complete set of transient spectra. It therefore appears to be very robust regarding changes in the repeating unit. Knowing this value, contactless determination of the polyfluorene film thickness has become possible by measuring the oscillation period in the transient spectra after photoexcitation. The transient circular dichroism kinetics shows the same type of periodic oscillation. This observation suggests that the CD spectrum also exhibits a pressure-induced shift, which is induced by the acoustic phonon. In addition, chirality changes in the cholesteric polymer through periodic modulation of the pitch length of the helical thin film stack might occur.
